# The risks of telehealth in radiation oncology: challenges and mitigation strategies

**DOI:** 10.3389/fonc.2025.1595975

**Published:** 2025-06-24

**Authors:** Sotiri Stathakis, Niko Papanikolaou, Jessica Ashford, Jason Stephens, Aaron LaRose

**Affiliations:** ^1^ Mary Bird Perkins Cancer Center, Baton Rouge, LA, United States; ^2^ The University of Texas Health Science Center at San Antonio, San Antonio, TX, United States

**Keywords:** telehealth, telemedicine, radiation oncology, risks, challenges, mitigation strategies, patient safety, cybersecurity

## Abstract

The integration of telehealth into radiation oncology represents a significant evolution in healthcare delivery, driven by the potential for enhanced patient accessibility, convenience, and improved multidisciplinary collaboration and operational efficiency. Telehealth modalities support a wide range of activities, from remote consultations and treatment planning discussions to on-treatment checks, toxicity management, and follow-up care. However, the successful adoption of telehealth is predicated upon the effective identification and management of substantial challenges. These include safeguarding sensitive patient data against cybersecurity threats, navigating the inherent limitations of remote clinical assessments, overcoming technological barriers that contribute to the digital divide, adhering to a complex and evolving regulatory and reimbursement environment, maintaining the crucial therapeutic patient-provider relationship, seamlessly integrating telehealth workflows into existing clinical operations, and upholding ethical principles, particularly concerning equitable access and algorithmic bias. While initial concerns about potential risks, such as increased rates of misdiagnosis or reduced patient satisfaction, were prominent during the early phase of rapid adoption, a growing body of recent empirical literature provides valuable insights into the actual impact of telehealth in radiation oncology and the effectiveness of various mitigation strategies. This paper offers a comprehensive and updated review of these multifaceted challenges, analyzing their potential influence on clinical outcomes, healthcare operations, and the patient experience. Drawing upon the latest evidence, it details proactive, evidence-based mitigation strategies – encompassing the strategic implementation of hybrid care models, investments in secure and user-friendly technological infrastructure, the development and adherence to standardized clinical and operational protocols, targeted training for providers and staff, and robust support mechanisms for patients. A structured, continuous risk management framework is presented as an essential component for navigating these complexities. By thoroughly understanding these challenges and actively implementing evidence-informed mitigation approaches, radiation oncology practices can successfully harness the considerable benefits of telehealth while rigorously protecting patient safety, ensuring the delivery of high-quality care, and promoting equitable access to cancer treatment for all individuals.

## 1 Introduction

Radiation oncology is a highly specialized discipline within cancer care, characterized by its intricate treatment planning, the use of sophisticated technology for precise radiation delivery, and the necessity for close monitoring of patients throughout their therapeutic journey. Effective radiation therapy requires a coordinated effort from a multidisciplinary team, including radiation oncologists, medical physicists, dosimetrists, radiation therapists, nurses, and support staff, alongside consistent engagement with the patient to manage side effects, address psychosocial needs, and ensure treatment adherence.

The global health crisis instigated by the COVID-19 pandemic necessitated rapid innovation in healthcare delivery models. Telehealth capabilities, encompassing a range of virtual technologies and services, were swiftly deployed across numerous medical specialties, fundamentally altering the modality of patient-provider interactions. Radiation oncology departments, facing the imperative to minimize non-essential in-person contact while maintaining critical cancer treatment schedules, rapidly adopted telehealth for activities such as initial consultations for certain disease sites, on-treatment virtual check-ins, multidisciplinary treatment planning conferences conducted remotely, and post-treatment follow-up appointmentP (1, 2) P. This pivot demonstrated the potential of telehealth to maintain access to care, reduce patient travel burdens (especially significant for oncology patients often experiencing fatigue or residing far from treatment centers), and enhance the efficiency of clinical operations.

Beyond the pandemic’s immediate pressures, telehealth offers compelling long-term prospects for radiation oncology. It can improve access to specialized care for patients in geographically remote or underserved areas, facilitate more frequent touchpoints for symptom management, streamline communication within the complex care team, and integrate with evolving digital health tools. These tools extend beyond simple video visits to include secure patient portals for messaging and data sharing, remote monitoring devices, and potentially AI-assisted applications for assessment and planning (3, 4).

However, the successful and sustainable integration of telehealth into the established workflows and clinical paradigms of radiation oncology demands a thorough understanding and proactive management of its inherent challenges and risks. These span a wide spectrum, from technical and operational hurdles to clinical, regulatory, legal, ethical, and interpersonal considerations. While the potential risks were acutely highlighted during the initial, rapid phase of adoption (5), a growing body of research now provides valuable insights into the actual impact of telehealth and the effectiveness of strategies developed to mitigate these concerns. This paper aims to provide a comprehensive, updated review of the critical challenges specific to telehealth implementation in radiation oncology, drawing upon recent literature to analyze their real-world effects and detailing evidence-based strategies for their effective mitigation. By addressing these multifaceted issues systematically, radiation oncology practices can harness the transformative potential of telehealth while ensuring patient safety, maintaining clinical excellence, promoting health equity, and fostering strong patient-provider relationships.

## 2 Risks, challenges, and mitigation strategies

The integration of telehealth into the complex environment of radiation oncology requires a deliberate and comprehensive strategy to address potential risks while preserving the high standards of patient safety and care quality. This involves balancing technological implementation with clinical best practices, regulatory compliance, and ethical considerations. The primary areas of challenge and evidence-informed strategies for their mitigation are detailed below.

### 2.1 Data security and privacy

- **Challenges:** Radiation oncology handles some of the most sensitive and comprehensive patient data, including detailed medical histories, diagnostic imaging studies (CT, MRI, PET), pathology reports, genomic data, and intricate treatment plans (including contours, dose distributions, and machine parameters). The digital nature of telehealth platforms and the transmission of this data over networks expose it to significant cybersecurity risks, including unauthorized access, data breaches, ransomware attacks, and identity theft (6, 7). A breach can have devastating consequences, leading to severe financial penalties, legal liabilities (especially under regulations like Health Insurance Portability and Accountability Act (HIPAA) in the United States or General Data Protection Regulation (GDPR) in Europe), operational disruptions, and significant damage to the institution’s reputation and patient trust (8). Integrating telehealth platforms with existing EHRs, treatment planning systems, and oncology information systems can create complex interfaces that introduce vulnerabilities if not meticulously secured and managed (9). Furthermore, the increasing reliance on cloud-based services and third-party vendors for hosting or managing telehealth data necessitates rigorous vetting and ongoing monitoring of their security practices (6).
- **Mitigation Strategies:**
- **Implement Robust Cybersecurity Infrastructure:** Deploy multi-layered security defenses, including next-generation firewalls, intrusion detection and prevention systems, and endpoint protection. Conduct regular vulnerability scanning and penetration testing to identify and remediate weaknesses proactively (7). Establish secure network segmentation for telehealth systems.
- **Mandatory Encryption:** Ensure all patient data is encrypted both in transit [using protocols like Transport Layer Security (TLS) and Secure Sockets Layer (SSL)] and at rest (on servers and devices) (10, 11). This protects data even if systems are compromised.
- **Strict Access Controls and Multi-Factor Authentication (MFA):** Implement role-based access controls (RBAC) to ensure users only have access to the minimum data necessary for their job function. Make MFA mandatory for all access to telehealth platforms and related patient data systems (6).
- **Regular Security Audits and Risk Assessments:** Conduct periodic, independent security audits and risk assessments specific to the telehealth infrastructure and integrated systems (10). Address findings promptly.
- **Comprehensive Compliance Program:** Maintain rigorous adherence to all relevant data protection laws and regulations, including HIPAA (11), GDPR, and state-specific privacy laws. This involves having clear, accessible privacy policies, obtaining necessary patient consents for data use, and establishing robust procedures for breach notification and response (12, 13).
- **Continuous Staff Training and Awareness:** Provide mandatory, ongoing cybersecurity training for all staff involved in telehealth. Training should cover recognizing and reporting phishing attempts, safe handling of patient data, password hygiene, and incident response procedures. Foster a culture of security awareness (10).
- **Rigorous Third-Party Risk Management:** Establish a formal process for evaluating the security posture of all telehealth vendors and third parties. Ensure contracts include stringent data security and privacy requirements, audit rights, and clear liability clauses (6).

### 2.2 Clinical effectiveness and assessment limitations

- **Challenges:** The clinical precision required in radiation oncology means that comprehensive patient assessment is vital for accurate diagnosis confirmation, treatment planning, monitoring response, and managing toxicities. Telehealth consultations, while convenient, inherently limit the ability to perform a full physical examination. This raises concerns about the potential to miss subtle but clinically significant findings, such as early signs of radiation dermatitis severity that may require dose adjustment, subtle changes in tumor sites accessible by palpation, or signs of complications like lymphedema or neuropathy (5). Early theoretical concerns posited that this limitation could potentially lead to misdiagnosis, delayed intervention, or suboptimal treatment outcomes (5).
- **Mitigation Strategies:**
- **Hybrid Care Model as Standard:** The most effective strategy is to adopt a **hybrid care model** that strategically combines virtual and in-person visits (2). This model leverages the strengths of telehealth for appropriate tasks (e.g., initial consultations for certain disease sites where imaging is primary, routine on-treatment check-ins for stable patients, follow-up appointments after treatment completion for surveillance) while reserving in-person visits for assessments requiring physical examination, complex symptom evaluation, procedures (e.g., simulation, biopsy), or when necessitated by patient preference or clinical instability (10).
- **Empirical Evidence of Safety:** It is crucial to base practice on the evolving empirical evidence. Recent large-scale studies provide reassurance regarding patient safety outcomes with telehealth integration in radiation oncology. The large cohort study by Cuaron et al. (14) is a prime example, demonstrating that the implementation of telehealth was not associated with increases in patient safety incidents, hospitalizations, emergency department visits, or delays in treatment completion in their cohort. This evidence helps to alleviate concerns that telehealth *inherently* leads to increased misdiagnosis or delays when implemented within appropriate clinical protocols.
- **Augmenting Virtual Assessments:** Enhance the information available during virtual visits. Encourage patients to send high-quality images or videos of visible areas of concern (e.g., skin reactions). Utilize remote monitoring devices for relevant physiological parameters if applicable. Ensure providers have immediate virtual access to all relevant patient data, including recent imaging, lab results, and previous treatment summaries (3).
- **Develop and Adhere to Standardized Protocols:** Create clear, disease-site-specific, or symptom-based protocols outlining when a telehealth visit is clinically appropriate, what minimum assessment is required during the virtual visit, and clear criteria for converting a virtual visit to an urgent or routine in-person appointment based on presenting symptoms or concerns (10).
- **Targeted Provider Training:** Provide specific training for radiation oncologists and clinical staff on how to conduct effective virtual assessments. This includes techniques for eliciting comprehensive patient-reported information, interpreting verbal cues and patient-provided visual information, and being acutely aware of the limitations of remote assessment and the triggers for recommending an in-person visit (10).

### 2.3 Technological barriers and digital divide

- **Challenges:** While technology is fundamental to telehealth, unequal access to necessary infrastructure and digital literacy skills represents a significant barrier to equitable care access. Reliable high-speed internet (broadband) is not universally available, particularly in rural and some urban areas (15, 16). Many patients, especially older adults, individuals with lower socioeconomic status, those with limited education, or individuals with disabilities, may lack access to appropriate devices (smartphones, tablets, computers) or possess the necessary digital literacy skills to effectively use telehealth platforms (15, 17). This digital divide can exclude vulnerable populations from benefiting from telehealth, potentially exacerbating existing disparities in cancer care access and outcomes (15). Beyond patient access, inconsistent software compatibility between different telehealth platforms, EHRs, and specialized oncology systems can lead to frustrating technical glitches, poor video/audio quality, frozen screens, or dropped connections during virtual appointments (18). These technical failures disrupt communication, consume valuable time, and can negatively impact the perceived quality of care and the therapeutic relationship.
- **Mitigation Strategies:**
- **Address Patient Access to Technology:** Implement programs to support patients who lack the necessary technology or internet access. This can include providing pre-configured, secure loaner devices (like tablets with integrated connectivity), partnering with community organizations to establish accessible telehealth sites, or providing financial assistance for internet access where feasible (2, 17).
- **Advocate for Infrastructure Investment:** Support and advocate for local, state, and federal initiatives aimed at expanding broadband internet access, particularly in underserved communities (17).
- **Provide Dedicated Technical Support:** Establish easily accessible technical support channels (phone, email, chat) staffed by individuals trained to assist both patients and providers with pre-visit technical checks and troubleshooting issues that arise during virtual appointments (2).
- **Prioritize User-Friendly Platforms:** Select telehealth platforms known for their intuitive design, ease of use across a range of devices and operating systems, and ability to function reasonably well even with moderate internet speeds. Standardizing on a limited number of platforms can reduce compatibility issues (2, 9).
- **Offer Digital Literacy Support and Education:** Provide clear, simple, and accessible training materials (e.g., short video tutorials, illustrated step-by-step guides, phone support walking patients through the process) to help patients become comfortable using the technology. Consider involving family members or caregivers in this training where appropriate.
- **Maintain Flexible Modalities:** While video visits are often preferred for communication cues, offer phone consultations as a standard backup option for patients who cannot connect via video, ensuring care continuity (18).

### 2.4 Regulatory and legal compliance

- **Challenges:** The regulatory environment governing telehealth is complex, fragmented, and continuously evolving (12, 13). Key challenges include navigating differing state licensing requirements for providers delivering care across state lines (13), which complicates providing care to patients who may travel or reside in neighboring states. Inconsistent and often opaque reimbursement policies from private payers and government programs (like Medicare and Medicaid) create significant financial uncertainty for radiation oncology practices, potentially limiting investment in telehealth infrastructure and training (12, 19). Keeping pace with updates to privacy laws, security regulations, documentation requirements, and prescribing rules for controlled substances delivered via telehealth adds substantial administrative burden and requires ongoing vigilance (13, 19). Beyond compliance, institutions face potential legal liabilities, including claims related to alleged misdiagnosis or delayed care resulting from perceived limitations of virtual visits, liability for data breaches, and responsibility for technical failures that directly impact patient safety (12).
- **Mitigation Strategies:**
- **Proactive Regulatory Engagement:** Actively participate in advocacy efforts with professional societies [e.g., American Society for Radiation Oncology (ASTRO), American Society of Clinical Oncology (ASCO)], state medical boards, and legislative bodies to promote the development of clear, consistent, and favorable telehealth regulations and reimbursement policies (2). Support initiatives like the Interstate Medical Licensure Compact.
- **Establish a Dedicated Compliance Function:** Designate a compliance officer or team responsible for staying current with all relevant telehealth regulations, ensuring internal policies and practices are aligned, and conducting internal audits (19).
- **Engage Specialized Legal Counsel:** Retain legal counsel with expertise in healthcare law, telemedicine, and data privacy to review telehealth policies, patient consent forms, business associate agreements with vendors, and potential liability exposures (12).
- **Implement Licensure Management Systems:** For practices providing care across state lines, utilize systems or processes to efficiently track and manage provider licenses in all relevant jurisdictions.
- **Standardize Billing and Reimbursement Processes:** Develop clear, standardized procedures for coding and billing telehealth services, train staff on these procedures, and proactively work with payers to understand their specific requirements and resolve reimbursement issues (11, 19).
- **Ensure Adequate Insurance Coverage:** Verify that institutional and individual provider malpractice insurance policies explicitly include coverage for telehealth services conducted within legal and professional guidelines (10).
- **Robust Documentation and Incident Response Planning:** Maintain thorough and accurate documentation for all telehealth visits (see Section 2.6), clearly noting the modality, attendees, key findings, and rationale for clinical decisions or the need for follow-up. Develop and regularly practice an incident response plan for cybersecurity breaches or adverse clinical events related to telehealth to mitigate legal risks and ensure a timely, appropriate response (10).

### 2.5 Patient-provider relationship and communication

- **Challenges:** A strong, trusting patient-provider relationship is fundamental to high-quality cancer care and treatment adherence. Concerns exist that the virtual nature of telehealth might hinder the development or maintenance of this relationship, potentially leading to patients feeling less personally connected, less understood, or that their care is less comprehensive compared to in-person interactions (18). Non-verbal communication cues, which play a significant role in building rapport and conveying empathy, can be more difficult to perceive and interpret effectively via video, and are completely absent in audio-only consultations (18). While many patients report high overall satisfaction with telehealth’s convenience, studies indicate varied preferences, with some patients expressing a preference for in-person visits, particularly for initial consultations, discussing difficult news, receiving complex explanations, or simply for the valued element of human connection and physical presence (20). Failing to acknowledge and address these preferences can impact patient experience and satisfaction.
- **Mitigation Strategies:**
- **Prioritize Video Consultations:** Strongly encourage and facilitate video visits over audio-only when technically possible for both parties, as video allows for visual cues that are vital for rapport and understanding (18).
- **Ensure Care Continuity:** Whenever feasible, schedule telehealth visits with the patient’s established radiation oncologist or a consistent member of their primary care team. Continuity builds trust and strengthens the relationship (18).
- **Train Providers in Virtual Communication Skills:** Provide specific training on best practices for virtual communication. This includes techniques like looking into the camera to simulate eye contact, active listening skills tailored for a virtual format, speaking clearly, ensuring a professional and distraction-free background, confirming patient understanding of complex information (using “teach-back” methods), and conveying empathy despite the physical distance (10).
- **Structure Visits for Connection:** Dedicate time at the beginning of the visit for non-medical rapport-building. Structure the clinical portion clearly but allow ample time for patient questions and concerns. Use plain language and avoid medical jargon.
- **Leverage Digital Tools to Enhance Understanding:** Utilize screen-sharing capabilities to review images or lab results together. Use or share digital educational resources (diagrams, videos) to help explain complex treatment concepts or symptom management strategies (18).
- **Actively Solicit and Act on Patient Feedback:** Implement post-telehealth visit surveys or follow-up calls to gauge patient satisfaction, comfort level with the technology, perceived quality of communication, and preferences for future visits. Use this feedback to continuously refine telehealth protocols and provider approaches (10).
- **Respect Patient Preference and Offer Hybrid Options:** Be sensitive to patient preferences regarding visit modality. Within the hybrid model, offer in-person visits when requested for specific reasons (e.g., sensitive discussions, feeling more comfortable) or when clinically indicated.

### 2.6 Workflow integration and documentation

- **Challenges:** Integrating a new modality like telehealth into established radiation oncology clinical workflows presents significant operational challenges. Processes for patient scheduling, virtual check-in, coordination with support staff, clinician assessment flow, integration of telehealth data into the EHR, and communication among the multidisciplinary team require substantial adaptation (9, 21). Technical interoperability challenges between disparate telehealth platforms, the core EHR, and specialized oncology systems often result in fragmented documentation, duplicated data entry, delays in accessing critical patient information (like real-time imaging or lab results), and inefficient communication pathways (9). These inefficiencies can slow down processes, increase the risk of errors or missed information, and add significant administrative burden on physicians, nurses, and support staff, potentially contributing to burnout (9, 22).
- **Mitigation Strategies:**
- **Comprehensive Workflow Analysis and Redesign:** Conduct a detailed analysis of current in-person workflows and design new, optimized workflows specifically for telehealth visits, considering patient check-in, provider flow, support staff roles, scheduling logic, and multidisciplinary team communication (9).
- **Prioritize Technical Interoperability:** Select telehealth platforms with proven, robust, and ideally bi-directional integration capabilities with the institution’s primary EHR and key oncology systems (e.g., oncology information system, treatment planning system) (2, 9). This minimizes manual data entry and ensures a unified patient record.
- **Standardized Documentation Templates and Protocols:** Develop clear, standardized templates within the Electronic Health Record (HER) or telehealth platform specifically for documenting telehealth visits. These templates should prompt inclusion of key information like the visit modality, attendees, consent obtained, summary of subjective and objective findings (noting limitations of virtual exam), assessment, plan, and instructions for follow-up (23).
- **Leverage Technology for Efficiency:** Utilize workflow-enhancing features within the telehealth or EHR system, such as automated appointment reminders, digital intake forms, secure messaging for team communication, and voice-to-text transcription for documentation assistance (9)].
- **Provide Thorough Staff Training and Define Roles:** Train all staff members involved in the telehealth process on the new workflows, platform usage, and documentation requirements. Clearly define roles and responsibilities for scheduling, technical support, patient education, pre-visit preparation, documentation, and post-visit follow-up (10).
- **Pilot Testing and Iterative Improvement:** Implement workflow changes through pilot programs in specific clinics or for particular patient types. Gather feedback from staff and providers to identify bottlenecks, inefficiencies, and technical problems, and use this information to iteratively refine the workflows and technical integration before expanding the program.

### 2.7 Ethical considerations and equity

- **Challenges:** Telehealth implementation must align with core ethical principles, particularly justice and equity (24). The digital divide represents a significant ethical challenge, as it can exclude vulnerable patient populations (those lacking technology, internet, or digital skills) from accessing care, potentially exacerbating existing healthcare disparities (15, 17). Ensuring truly informed consent for telehealth visits is ethically crucial; patients must fully understand the nature of the service, including its differences from in-person care, potential limitations (like physical exam), privacy risks, and their right to choose an alternative modality (24). Furthermore, the increasing use of artificial intelligence (AI) and machine learning algorithms within telehealth or integrated decision support systems in oncology introduces the risk of algorithmic bias (25). If these algorithms are trained on biased data or are not validated across diverse patient populations, they could inadvertently lead to differential treatment recommendations or outcomes based on race, socioeconomic status, age, or other factors, raising serious ethical concerns about fairness and non-maleficence (25).
- **Mitigation Strategies:**
- **Prioritize Health Equity and Access:** Design telehealth services with an explicit focus on equitable access. This includes actively working to bridge the digital divide (as discussed in Section 2.3) by providing technology support and digital literacy training, offering multilingual support and translation services, and ensuring platforms are accessible for individuals with disabilities (2, 15, 17).
- **Develop Robust, Accessible Informed Consent:** Create clear, comprehensive, and easily understood informed consent processes specifically for telehealth (23, 24). Materials should explain the benefits, risks, technical requirements, privacy considerations, and limitations of virtual care using plain language and offering various formats (written, video, verbal explanation). Explicitly state the patient’s right to choose an in-person visit.
- **Address Algorithmic Bias and Ensure Transparency:** If utilizing AI or algorithmic tools, implement stringent processes for bias detection and mitigation (25). This involves ensuring training data is diverse and representative, regularly auditing algorithm performance across different demographic groups, and maintaining transparency about how AI tools are used in clinical decision-making. Crucially, human oversight by trained clinicians should be maintained, especially for critical decisions (25).
- **Establish Ethical Oversight and Governance:** Form an institutional ethics committee or a dedicated telehealth ethics working group to provide ongoing guidance, review policies, and address ethical dilemmas related to telehealth implementation, particularly concerning equity, consent, and the use of AI (24).
- **Collect and Analyze Equity Data:** Systematically collect data on telehealth utilization demographics to identify potential disparities and target interventions to reach underserved populations more effectively (15).

## 3 Comprehensive risk management framework

Implementing and sustaining telehealth safely and effectively in radiation oncology requires more than addressing individual challenges; it demands a systematic, proactive, and continuous risk management framework. This framework should be integrated into the department’s overall quality assurance and patient safety programs and involve input from all stakeholders, including patients, providers, technical staff, and administrators. The core components of such a framework include:

1. **Risk Identification:** Proactively identify potential risks across all domains: clinical (e.g., assessment limitations), technical (e.g., platform reliability, cybersecurity), operational (e.g., workflow disruptions), regulatory/legal (e.g., compliance issues, liability), financial (e.g., reimbursement shortfalls), ethical (e.g., equity), and human factors (e.g., provider burnout, patient discomfort). This should be an ongoing process, incorporating feedback from staff and patients, incident reports, and scanning the external environment for emerging threats or changes in regulations.
2. **Risk Assessment:** Evaluate the likelihood of each identified risk occurring and the potential severity of its impact on patient safety, clinical outcomes, operational efficiency, financial stability, and reputation. Use a structured approach (e.g., a risk matrix) to prioritize risks based on their potential impact and likelihood.
3. **Risk Mitigation:** Develop and implement targeted strategies to reduce the probability or impact of prioritized risks, drawing from the detailed strategies outlined in Section 2. Mitigation efforts should be evidence-based and tailored to the specific context of radiation oncology.
4. **Risk Monitoring:** Continuously monitor the effectiveness of implemented mitigation strategies and key performance indicators (KPIs) related to telehealth safety, quality, efficiency, satisfaction, and compliance. Examples include tracking rates of technical issues, cybersecurity incidents, patient satisfaction scores, clinical outcomes for telehealth patients, rates of conversion from virtual to in-person visits, and successful billing rates.
5. **Risk Review and Improvement:** Periodically review the entire risk management process, analyze monitoring data, investigate any incidents or near misses related to telehealth, and gather feedback. Use this information to refine risk assessments, update mitigation strategies, improve protocols, and adapt workflows as technology, regulations, and practice patterns evolve (10). This iterative process ensures that the telehealth program remains robust and responsive.

Embedding this framework within existing quality improvement and safety structures (e.g., morbidity and mortality conferences, peer review, accreditation requirements like those from the American College of Radiology or ASTRO) helps to ensure that telehealth risks are addressed systematically as part of routine clinical governance (10).

## 4 Discussion and future directions

Telehealth has undeniably reshaped the landscape of healthcare, and its integration into radiation oncology presents unique opportunities and responsibilities. This subspecialty requires a finely coordinated, multidisciplinary effort, often over an extended treatment period. Virtual modalities, when integrated carefully, can enhance patient access to subspecialized radiation oncology services (e.g., stereotactic body radiation therapy, brachytherapy, or proton therapy) for patients in geographically remote or resource-limited settings. This is particularly relevant for community cancer centers and rural regions that lack local expertise in advanced modalities.

While early concerns regarding missed toxicity assessments or diminished therapeutic rapport were valid, recent studies—such as Cuaron et al. (14)—indicate that thoughtful integration of telehealth does not compromise patient safety or treatment completion timelines. In radiation oncology, these findings support the continued use of telehealth for specific tasks such as contour review conferences, routine follow-ups (e.g., for prostate or breast cancer where toxicities are predictable and manageable remotely), and multidisciplinary tumor boards. When these functions are shifted to telehealth, they can reduce the in-person clinic burden, free up on-site resources for patients requiring hands-on care, and enhance care team coordination, especially across multiple campuses.

Notably, the impact on patient-provider communication is a nuanced issue in this field. Radiation oncologists often develop longitudinal relationships over 4–7 weeks of daily treatment (20). Virtual check-ins—when used selectively—can preserve that continuity while reducing fatigue from travel. However, first-time consultations for complex cancers (e.g., head and neck, gynecologic malignancies) may still benefit from in-person assessments to ensure optimal clinical planning, including mask fabrication, simulation, and target delineation.

To ensure radiation oncology continues to lead in patient-centered care delivery, it is essential that telehealth implementation focuses on disease-site-specific standards, such as using video visits to monitor skin reactions in breast cancer or mucositis in head and neck cancer patients. Similarly, virtual survivorship care may be particularly well-suited to diseases like prostate cancer, where long-term follow-up focuses on PSA monitoring, urinary or bowel function, and quality-of-life assessments—all of which can be discussed and tracked remotely with high patient satisfaction (1).

Looking forward, key priorities include validating AI-assisted tele-triage tools tailored for radiotherapy patients (e.g., tools that detect early signs of radiation-induced toxicity via patient-submitted images or symptom checklists) and incorporating remote dosimetry consults for geographically distributed teams (3). Additionally, development of telehealth protocols for managing emergent on-treatment symptoms—such as managing early-onset radiation pneumonitis remotely with pharmacy integration—could prevent unnecessary treatment interruptions.

Future research should explore how hybrid telehealth models impact the total duration of radiation therapy episodes, rates of missed treatments, and early toxicity intervention. For example, can virtual check-ins with embedded PROMs (Patient-Reported Outcome Measures) lead to earlier detection of toxicities such as gastrointestinal upset during pelvic RT, thereby reducing unplanned breaks? Economic evaluations specific to the cost of unplanned travel, time off work, or caregiver burden in radiation patients could further define the financial benefits of targeted virtual interventions (22, 23).

Policy advocacy must remain focused on ensuring telehealth reimbursement models in oncology reflect the value of procedural and planning time, not just face-to-face interactions. For example, remote physics QA checks, virtual peer reviews, and tele-dosimetry planning reviews are integral to quality patient care but are rarely captured in current billing structures.

By embedding telehealth within a clearly defined, site-specific, and role-appropriate framework, radiation oncology can transform the delivery of high-quality cancer care without compromising safety or efficacy—and in many cases, while improving access and patient-centeredness.

## 5 Conclusion

Telehealth offers transformative potential for radiation oncology, promising enhanced patient access, improved convenience, and increased operational efficiency. However, its successful and safe implementation necessitates careful consideration and proactive management of significant risks spanning data security, clinical assessment, technology, regulation, patient relationships, workflows, and ethics. While early concerns about issues like misdiagnosis and patient satisfaction were prominent, recent empirical evidence provides reassurance that these risks are manageable. By adopting comprehensive mitigation strategies – including implementing robust cybersecurity, embracing hybrid care models supported by evidence (14), investing in accessible technology, standardizing protocols (10), training providers, and prioritizing equitable access (17) – radiation oncology practices can effectively address these challenges. A structured risk management framework is essential for continuous monitoring and improvement. By diligently addressing potential pitfalls and leveraging evidence-based approaches, radiation oncology stakeholders can successfully integrate telehealth as a powerful tool to maintain high standards of patient safety and care quality, improve access to essential treatments, and shape the future of cancer care delivery. Continued research and policy evolution will further optimize telehealth’s role in this critical field.
